# Nurses and Climate Change: A Narrative Review of Nursing Associations’ Recommendations for Integrating Climate Change Mitigation Strategies

**DOI:** 10.1177/08445621241229932

**Published:** 2024-02-19

**Authors:** Coralie Gaudreau, Laurence Guillaumie, Édith Jobin, Thierno Amadou Diallo

**Affiliations:** 1 Faculty of Nursing Sciences, Public/Community Health Programs, Université Laval, Quebec City, Quebec, Canada; 2 CHU de Québec–Université Laval Research Centre, Québec, Québec, Canada

**Keywords:** Climate change, environmental issues, nurses’ associations, nursing practice

## Abstract

**Background:**

According to the World Health Organization, climate change is the greatest challenge of the twenty-first century. It is already affecting the health of many Canadians through extreme heat, wildfires and the expansion of zoonotic diseases. As trusted professionals, nurses are in favourable position to take action on climate change.

**Purpose:**

To document the recommendations issued by Quebec, Canadian, American and international nursing associations regarding nursing practices that address climate change or environmental issues.

**Methods:**

This narrative review was conducted by establishing a list of environmental and general nursing associations in the geographical areas of interest through Google searches as well as by retrieving documents about climate change or environmental issues published by these organizations on their websites. Data related to the documents’ characteristics and recommended nursing roles were then extracted.

**Results:**

The review identified 13 nurses’ organizations and 20 documents describing 37 recommendations for nurses in seven socioecological areas: individual, patient-focused, workplace, nursing associations, public health organizations, political and education.

**Conclusions:**

There is a gap between the breadth of roles that nurses may be called upon to play in addressing climate change and the degree to which relevant organizations are prepared to create the required conditions for them to do so. Several lessons emerged, including that the urgency of the climate crisis requires clear guidelines on how nurses can integrate climate change and its resultant health concerns into practice through nurses’ associations, education and bottom-up nursing innovations. Funding is required for such initiatives, which must also prioritize health inequalities.

## Background and purpose

In Canada, health costs related to climate change are currently estimated at several billion dollars per year—an amount that is expected to skyrocket by 2100 due to heat and smog, lost quality of life and premature death (Canadian Institute for Climate Choices, 2021). The World Health Organization (WHO) conservatively estimates that climate change will cause approximately 250,000 additional deaths annually between 2030 and 2050 due to malnutrition, malaria, diarrhea and heat stress (WHO, 2021). In fact, climate change is already affecting the physical health and well-being of people around the world (Pörtner et al., 2022). According to the World Meteorological Organization, nearly 9% of the world's population is undernourished, partly due to climate variability and extreme temperatures (WMO, 2021). Weather-related events displaced 23 million people annually from 2010 to 2019 (WMO, 2021). Air pollution is responsible for 7 million deaths per year and is considered the fourth leading risk factor for premature death (Health Effects Institute (HEI), 2020; WHO, 2021). Furthermore, 2020 was one of the three warmest years on record with a global mean temperature 1.2°C above that of the pre-industrial era (World Meteorological Organization, 2021). A temperature increase of 3°C is expected by the end of this century, far exceeding the 2015 Paris Agreement target to limit global warming to less than 2°C, and ideally 1.5°C, above pre-industrial levels (UN Environment Programme, 2020). In Canada specifically, the temperature is increasing at twice the rate of global warming (Government of Canada, 2019). According to a recent Health Canada report, climate change is already affecting the health of Canadians through rising temperatures, extreme heat, wildfires and the expansion of zoonotic diseases, such as Lyme disease (Berry et al., 2022).

Some authors have singled out nurses as particularly well placed to take action on climate change; nurses are trusted by the public (Kurth, 2017) and have a duty to educate patients and to protect population health (Kangasniemi et al., 2014; Levin & Chandler, 2018), as well as making up a large workforce. Nurses could set an example on how to tackle climate change (Maibach et al., 2019), including by helping to make the healthcare system itself more environmentally sustainable (Muñoz, 2012). Although nurses are still new at this work, some recent advancements are worth considering as we conceptualize nurses’ potential contributions to fighting climate change. Two nursing models analyze health from an environmental perspective: the WE ACT PLEASE model (Schenk, 2019), a framework for environmental stewardship in nursing describing different domains of pollution of the health-care system, the level of actions of nurses and key professional elements to guide nurses in decreasing health-care pollution and Earth-Caring, an ecocentric values-based caring framework that address climate change, health impacts and the needs of all forms of life (Anderko et al., 2014). Additionally, a few models have been used to underline the relevance of involving nurses and other health professionals in environmental issues, including the One Health approach, which calls for increased collaboration between academics, policymakers and citizens to solve problems related to climate change; nurses could be included in this collaboration (WHO, 2017; Zinsstag et al., 2018). Some also suggest the Ecological Planetary Health Model to show how nurses could take up a leadership role with regard to learning, teaching and communicating about planetary health, achieving environmental sustainability and human well-being and proposing a framework for the inclusion of climate change in nursing education curriculum or professional education (Kurth, 2017; Leffers et al., 2017). Finally, the United Nations Sustainable Development goals can be used to establish the role of the nursing profession in addressing climate change (Lilienfeld et al., 2018). Overall, these models support a systemic, inclusive, sustainable and planetary view of health (Anderko et al., 2014; Kurth, 2017; Lilienfeld et al., 2018).

A few studies have explored nurses’ perceptions of their professional role in addressing climate change issues (Ordan et al., 2018; Tiitta et al., 2021) and highlighted some facilitating factors and barriers to addressing climate change in their practice. Indeed, adopting a planetary health perspective has been highlighted in previous studies as a facilitating factor to increase nurses’ knowledge of climate change and integrate it into education and research (Kalogirou et al., 2020; Rosa & Upvall, 2019; Tiitta et al., 2021). Reported barriers include nurses’ lack of knowledge on climate change and on how it is related to nursing practice (Leffers et al., 2017; Ryan et al., 2020; Tiitta et al., 2021), resistance to integrating it into nursing school curricula (Leffers et al., 2017), a lack of time in everyday practice (Ryan et al., 2020) and a lack of initiatives promoting environmentally responsible nursing practices (Anaker et al., 2015; Kalogirou et al., 2020). Nurses have consistently recommended increasing the profession's knowledge of climate change and healthcare through postsecondary and continuing education (Kalogirou et al., 2020; Numminen et al., 2015; Tiitta et al., 2021). Although nurses recognized that extreme weather impacts patients’ health outcomes (Tiitta et al., 2021), climate change was largely reported to be a personal rather than a professional concern (Kalogirou et al., 2020). In short, nurses’ current and potential roles in addressing climate change remain vague (Kalogirou et al., 2020). Some nurses have therefore called for clarification on their role in managing climate change issues (Kalogirou et al., 2020) and tools to support their integration of this new role into their everyday practice (Polivka et al., 2012).

Some authors have begun formulating recommendations for nurses’ role regarding climate change. Their recommendations include creating committees to help implement protocols, strategies or guidelines for greener care (Lilienfeld et al., 2018; Sayre et al., 2010) and assessing the environmental risks and needs of patients, particularly the most vulnerable (Leffers et al., 2017; Levin & Chandler, 2018). Some nursing associations have initiated projects addressing climate change issues and care. For example, the Alliance of Nurses for Healthy Environments (ANHE) developed the Work-Home-Adaptation-Mitigation (WHAM) Grid, which describes actions that nurses can perform personally and professionally to fight climate change (Cook et al., 2019). Similarly, Martin and Vold (2019), in a publication from the Canadian Federation of Nurses Unions, provide 26 recommendations for what the profession can do to combat climate change (e.g., work collaboratively with employers to green workplaces, educate patients about climate change, and promote active transportation and sustainable diets). To the best of our knowledge, there has not yet been a review of nursing associations’ perspectives on the role of nurses in addressing climate change. Such a review could provide an overall picture of the initiatives and innovations required to support nurses in this role.

As such, the aim of this review was to document the recommendations issued by Quebec, Canadian, American and international nursing associations regarding nursing practices intended to address climate change and other environmental issues. The specific objectives were as follows: 1) to describe the characteristics of such recommendations, 2) to provide a summary of the recommendations and a classification according to their socioecological area, and 3) to draw lessons from these recommendations.

## Methods and procedures

### Design

A narrative review was chosen for its ability to document new and emerging practices and to summarize all types of evidence, including grey literature (e.g., institutional reports) (Baethge, 2019). A narrative review addresses a specific research question and provides a summary of included studies, without a systematic literature search (Baethge, 2019). Narrative reviews have been extensively used in previous studies to set out syntheses of clinical guidelines (Bruinsma et al., 2016; Hayes et al., 2018) and to review nurses’ roles or practices (Biswas et al., 2019; Hulse, 2022; Vasiloglou et al., 2019). The Scale for the Assessment of Narrative Review Articles checklist was used to guide the present study (Baethge, 2019).

### Research strategy

The literature search was conducted in May 2020 and updated in March 2022 using Google and a two-step process. First, a Google search was conducted to establish a list of general and environmental nursing associations operating provincially in Quebec, nationally in Canada and the United States, and internationally. Quebec's nursing associations were singled out from Canada's associations since the authors of this article are located in this province and therefore wanted to document the recommendations specific to nurses from this area, if any. A combination of keywords related to nursing associations, climate change and environment, and the geographic areas of interest were used in English and French. Second, a Google search was conducted with the list of identified nursing associations and keywords related to climate change, sustainability, environmental issues and greening to identify relevant documents. In addition, the websites of these associations were searched using similar keywords and additional documentation was requested from these organizations via email. This two-step process made it possible to highlight the proportion of nurses’ associations that have published documents on nurses’ role in relation to climate change.

### Inclusion and exclusion criteria

Several inclusion and exclusion criteria were used. To be included, a document had to be published or authored by a general or environmental nurses’ association. Documents produced by discipline-specific nursing associations (e.g., an oncology nurses’ association) or by nursing educators or students’ associations were excluded. The nurses’ associations had to operate provincially in Quebec, nationally in Canada or in the United States, or internationally. These geographic areas were chosen for a larger research project intended to promote Quebec nurses’ involvement in environmental and climate change issues, of which this study constitutes a part. All types of documents related to environment or climate change issues (e.g., position statements, articles in professional journals, reports, and web pages or articles) were included. Documents that did not meet these criteria were excluded from the review.

### Data extraction

Data extraction was performed collaboratively by two team members (CG, LG). The Excel extraction form and extraction manual was pilot-tested and amended prior to beginning the full review. Data extracted pertained to document characteristics (authors, year, type of document, type of nursing association, country, main objective) and recommendations regarding nurses addressing environmental or climate issues. A recommendation was broadly defined as any action, practice or commitment expected of nurses. Recommendations were coded using complete sentences from the documents without reformulation.

### Data synthesis

A thematic analysis was conducted on the qualitative data extracted. A mixed inductive and deductive approach was used to develop the codes. Two team members developed a first version of the codebook from the data and a list of recommendations issued by the Canadian Federation of Nurses Unions, which was chosen for its large number of recommendations (Martin & Vold, 2019). After summarizing the data from each code, two team members formulated recommendations representative of each code.

## Results

A total of 13 nurses’ organizations were identified for our chosen geographical areas (two organizations in the province of Quebec, four federal ones in Canada, five federal ones in the US, and two international). Almost two thirds (n = 8) of these organizations had published documents describing nurses’ anticipated roles in tackling environmental and climate change issues, for a total of 20 documents (see Table 1). Of these 20 documents, 11 were reports, five were position statements, and four were articles or website pages published on the organization's website. Most documents were published by general professional nurses’ associations (n = 9) or environmental nurses’ associations (n = 9). One document was written by a professional order (n = 1). Four documents were published between 2005 and 2009 and nine documents were published between 2015 and 2019. No publication date was provided for the remaining six documents. Seven documents were published by national Canadian associations, ten by national associations in the United States, two by international organizations, and one by a Quebec association.

**Table 1.** Descriptive characteristics of included documents.

Characteristics		Number of documents (n = 20)
Years	2005–2009	4
	2010–2014	0
	2015–2019	9
	2020–present	1
	Undated	6
Type of nursing organization	Orders	1
	Unions	1
	Professional associations (general)	9
	Associations dedicated to the environment	9
Organization's geographic area of influence	Quebec (provincial)	1
	Canada (national)	7
	United States (national)	10
	International	2
Type of publication	Web article	4
	Position statement	5
	Report	11
Number of recommendations in the document	1–5	12
	6–10	5
	11–15	2
	16–20	0
	21+	1

Of these 20 documents, less than half offered more than five recommendations (n = 9). Only one document provided more than 15 recommendations (Martin & Vold, 2019). The objectives of these documents included mainly the following: 1) to inform and educate nurses about the impacts of climate change on the health of the population, 2) to discuss the role of nurses in environmental health and 3) to equip nurses for climate change (e.g., by developing a conceptual framework, expected roles, actions, strategies, advice or principles to guide nurses in managing climate change issues) (see Table 2).

**Table 2.** Summary of included documents.

Associations^1^	Number of documents published by the association	Document title	Identification number	Year	Main objective	Type of publication
**Quebec**						
Order of Nurses of Quebec (OIIQ)	1	Climate Change Impacts on Population Health and Nursing Practice	1	2019	Raise awareness about nurses’ obligation to assess, monitor, and teach the population about climate change and encourage vigilance with vulnerable clients	Position statement
**Canada**						
Canadian Nurses Association (CNA)	6	Nurses and Environmental Health	2	2017	Discuss the role of nurses in environmental health	Position statement
		Climate Change and Health	3	2017	Discuss the role of nurses in climate change adaptation and action	Position statement
		Framework for the Practice of Registered Nurses in Canada	4	2015	Provide a framework to promote a common understanding of registered nurses’ practice, competencies and contributions among Canadian nurses, students and stakeholders	Report
		The Role of Nurses in Greening the Health System	5	2008	Support and enhance the contribution of nurses in reducing the environmental impact of the health system	Report
		The Role of Nurses in Addressing Climate Change	6	2008	Provide information on climate change and its impact on health as well as identify nurses’ role in climate change adaptation and strategies	Report
		The Environment and Health: An Introduction for Nurses	7	2007	Identify links between health and environment, explore nurses’ role and identify ways to integrate environmental health practices into nursing, education, research and policies	Report
Canadian Federation of Nurses Union (CFNU)	1	Climate Change and Health	8	2019	Provide a resource on the links between climate change and health and recommendations that could be supported by health organizations	Report
**United States**						
Alliance of Nurses for Healthy Environments (ANHE)	6	Nurses Drawdown: Take Action	9	2020	To encourage nurses from all specialties and practice settings to take action in five key areas: energy, food, mobility, gender equity and nature	Web article
		Climate and Health Toolkit: Disaster preparedness	10	2018	Describe actions that nurses can take during environmental disasters	Web article
		Climate Change, Health, and Nursing: A Call to Action	11	2017	Explore regional differences in climate change and discuss nurses’ role in climate change adaptation and mitigation strategies	Report
		Climate and Health Toolkit: Communications	12	Undated	Discuss nurses’ duty to include climate change in their communications	Web article
		Climate and Health Toolkit: Policy and Advocacy	13	Undated	Discuss the role of nurses in advocating for change to create a healthier environment	Web article
		Getting Started with Climate Solutions: A Guide for Nurses	14	Undated	Provide a guide for nurses to contribute to climate change action by reducing their own impact and encouraging individuals to act	Report
Nurses Climate Challenge	3	Taking action on climate change: A guide for health professionals at work and home	15	Undated	Describe actions that can be taken by health professionals to make a positive impact and reduce the negative impacts of climate change	Report
		Recommendations for your hospital administration	16	Undated	Discuss recommendations nurses can make to hospital administrations	Report
		How to get started as a nurse advocate for climate policy solutions	17	Undated	Provide resources for nurses advocating for climate policies and on how to influence public opinion	Report
American Nurses Association (ANA)	1	ANA's Principles of Environmental Health for Nursing Practice with Implementation Strategies	18	2007	Provide principles for environmentally friendly care and implementation strategies, as well as argue for nurses as environmental health activists	Report
**International**						
*Sécretariat international des infirmières et infirmiers de l’espace francophone* (SIDIIEF; International secretariat of Francophone nurses)	1	*Infirmières et infirmiers à l’avant-garde d’une planète en santé*	19	2019	Take position on the health consequences of environmental issues, propose recommendations and affirm nurses’ commitment to environmental health	Position statement
International Council of Nurses (ICN)	1	Nurses, climate change and health	20	2018	Discuss nurses’ potential contributions to climate change mitigation	Position statement
						

Note: ^1^20 documents were published by a total of eight nursing organizations, out of 13 nurses’ organizations identified within the geographical categories used in this article. The five organizations that were identified in this article but did not publish on the topic were the *Association Québécoise des Infirmières et Infirmiers* (AQII) for Quebec; for Canada, the Canadian Coalition for Green Health Care and the Canadian Association of Nurses for Environment (CANE); and for the United States, Health Care Without Harm and National Nurses United.

A total of 37 different recommendations were identified. These recommendations relate to seven socioecological areas: 1) individual, 2) patient-focused, 3) workplace, 4) nursing associations and unions, 5) departments of health and public health organizations, 6) political action, and 7) post-secondary and continuing nursing education. The recommendations were most frequently patient- (n = 17) and worksite-focused (n = 15). The most cited recommendations (11 each) were to provide general information on climate change and pro-environmental practices to patients (included in documents #2, 5, 6, 8, 10, 12, 14, 15, 17, 18, 20; see associated references in Table 3) and to work with their employers to reduce greenhouse gas emissions and green the workplace (1, 3, 4, 7, 8, 11, 14, 15, 16, 18, 20).

**Table 3.** Synthesis of recommendations according to socioecological area and number of documents supporting each.

Recommendations	Number of documents supporting recommendation (identification numbers)
***Individual***	
Adopt personal practices that support the environment and reduce GHG emissions	7 (1, 2, 3, 9, 11, 14, 15)
Learn about the impacts of climate change on health as well as solutions	5 (1, 11, 14, 17, 18)
Support and engage with community-based climate change initiatives	4 (1, 6, 9, 15)
Participate in research on environmental health issues	2 (2, 11)
Collaborate with other professionals and experts to recognize climate change as a global emergency	2 (1, 2)
***Patient-focused***	
Provide general information on climate change and pro-environmental practices	11 (2, 5, 6, 8, 10, 12, 14, 15, 17, 18, 20)
Provide specific advice to patients, including by e-mail and telephone, on health issues related to climate change that may affect them (extreme temperatures, poor air quality, zoonotic diseases) and on adaptation measures	6 (1, 3, 5, 7, 8, 11)
Encourage active transportation, carpooling and public transportation	4 (5, 8, 9, 14, 20)
Determine individuals’ vulnerability to climate change, including psychological impacts	3 (2, 8, 13)
Recommend a healthy, sustainable diet (e.g., reducing consumption of red meat)	3 (8, 9, 14)
Assess environmental risks and inform communities, particularly vulnerable or at-risk groups	1 (2)
***Workplace***	
Work with employers to reduce GHG emissions and green the workplace	11 (1, 3, 4, 7, 8, 11, 14, 15, 16, 18, 20)
Form green teams to promote and implement greener practices in the workplace	5 (7, 8, 11, 15, 20)
Prepare for the most likely climate change risks in the area and who they will most impact (evacuation, emergency response protocols)	4 (8, 10, 15, 16)
Encourage hospital staff to use public transportation, carpooling or active transportation	5 (8, 9, 11, 14, 15)
Encourage sustainable food services	4 (8, 11, 15, 16)
Encourage the use of renewable energy in the facility	2 (8, 15)
Use programmable thermostats and adjust office thermostats to save energy, turn off lights in unoccupied rooms, use natural lighting, and unplug appliances or electronics when not in use	2 (14, 15)
Encourage procurement teams to purchase products with a lower environmental impact	3 (6, 9, 18)
Encourage membership in the Canadian Coalition for Green Healthcare and the use of their tools	1 (8)
Encourage pension divestment from high-emitting sectors and investment in clean technology and low-emitting sectors	1 (8)
Promote video conference meetings	1 (15)
Request forums where colleagues can discuss climate change and get informed	4 (6, 12, 14, 15)
***Nurses’ associations or unions***	
Encourage position papers, events and public statements supporting policies in favour of reducing GHG emissions and tackling climate change issues	7 (3, 5, 6, 7, 11, 18, 20)
Support the inclusion of ecological determinants of health and knowledge of climate change in training and curricula	1 (8)
Join nurses’ associations for the environment	1 (17)
***Departments of health and public health organizations***	
Advocate for a more sustainable economy and the reduction of GHGs	4 (5, 8, 11, 19)
Promote policies that recognize the urgency of climate change and advocate for integrating solutions into policy	3 (1, 3, 14)
Identify the populations and geographic areas most vulnerable to climate change	3 (1, 8, 20)
Collaborate with other professionals and experts to promote monitoring of current and future threats	1 (8)
Provide training in environmental impact assessment	1 (8)
***Political***	
Write open letters and use social media to influence opinion and political authorities	6 (2, 8, 11, 13, 14, 17)
Support the transition from fossil fuels to renewable energy	1 (8)
Support the carbon tax as a lever for behavioural change in society and for recognizing the true economic and health costs of air pollution and GHGs	1 (8)
Advocate for a fair and equitable transition for major GHG-emitting sectors	1 (8)
***Post-secondary and continuing nursing education***	
Provide initial and continuing education on climate change	4 (8, 11, 19, 20)
Develop intervention skills in the context of extreme weather events, epidemics, climate refugees and people experiencing psychological distress related to climate change and integrate such skill development into training programs	1 (8)

Note: GHG = greenhouse gas.

For nurses’ associations and unions, it was recommended that nurses highlight environmental and climate issues in communications (n = 7) (3, 5, 6, 7, 11, 18, 20). Personal and individual recommendations were that nurses should learn about climate change (n = 5) (1, 11, 14, 17, 18), adopt an eco-responsible lifestyle (n = 7) (1, 2, 3, 9, 11, 14, 15) and get involved in community projects (n = 4) (1, 6, 9, 15). For departments of health and public health organizations, recommendations included advocating for these institutions to acknowledge the urgency of climate change and integrate solutions into governmental and public initiatives (n = 3) (1, 3, 14), in addition to advocating for a more sustainable economy and the reduction of greenhouse gas emissions (n = 4) (5, 8, 11, 19). Political recommendations included writing open letters and using social media to influence opinion and political authorities (n = 6) (2, 8, 11, 13, 14, 17). Finally, it was recommended that climate change and its impacts be integrated into post-secondary and continuing nursing education (n = 4) (8, 11, 19, 20). The importance of providing training on nursing care and extreme weather events, epidemics, climate refugees and people experiencing psychological distress related to climate change was mentioned once (8).

## Discussion

This narrative review aimed to document the recommendations issued by nurses’ associations from Quebec, Canada and the United States, as well as international associations, for targeting environmental and climate change issues. Of the 13 nurses’ organizations identified, five had not published any kind of documents about climate change. This highlight that several nurses’ associations still need to take action and provide recommendations to their members, especially considering that the importance of nurses’ role regarding climate change is well documented and that nurses are asking for a clarification of their role (Kalogirou et al., 2020; Polivka et al., 2012). More than half of the included documents (n = 11) contained fewer than six recommendations and only one document contained more than 15 (Martin & Vold, 2019). These numbers emphasize that some documents may not cover the full scope of actions that nurses could include in their practice. Recommendations were extremely diverse and required a wide range of knowledge, skills, and guidelines or tools to support their implementation. Overall, results showed modest leadership from nurses’ associations on climate change. Four main lessons can be drawn from these results: nursing innovations that address environmental and climate change issues need financial support; current recommendations do not fully consider health inequities; nurses’ roles in tackling environmental and climate change issues have received little attention from nurses’ associations; and we must clarify how nursing education can contribute to addressing environmental and climate change issues. In the text that follows, we discuss each of these lessons in detail.

First, nursing innovations that address environmental and climate change issues need financial support. Our review brought to light 37 different recommendations for nurses in seven socioecological areas. However, financial support is required to develop such a wide range of skills. Therefore, health and research agencies, government and public organizations, and practice settings should make financial incentives available. Grants were reported to accelerate the systemic integration of green, resilient and inclusive practices within health-related policies (IPCC, 2014), as well as favouring new project development, changes in practice, and intersectoral collaboration between stakeholders (Government of Canada, 2019).

Second, the recommendations in these documents do not sufficiently consider health inequities. Although nurses may provide care for people vulnerable to climate change (Butterfield et al., 2021), fewer than half of the documents (n = 8) addressed these issues. Health inequities were addressed in three recommendations: to assess environmental risks and inform communities, particularly vulnerable or at-risk groups (included document #2, see complete reference in Table 2); to prepare for the most likely climate change risks in your area and who these risks will most impact (8, 10, 15, 16); and to identify the populations and geographic areas most vulnerable to climate change (1, 8, 20). However, there is significant evidence that climate change increases health inequities, resulting in additional costs to the health care system (Canadian Institute for Climate Choices, 2021). People who are socially marginalized or living in poverty are most likely to be negatively affected by climate change in terms of their physical and mental health, including premature death (IPCC, 2014). A study has shown that in 70% of US cities, people living below the poverty line were more exposed to urban heat islands than people living two times above the poverty line (Hsu et al., 2021). In this study, the recommendations addressing health inequities pertained to two socioecological areas: patient care and health departments or public health organizations. However, previous studies have highlighted other areas of action, such as advocating for policies addressing health inequities in relation to climate change (LeClair et al., 2021; Simmonds et al., 2021), developing infrastructure to aid people before they become impoverished (Lilienfeld et al., 2018), and collaborating with international policymakers (LeClair et al., 2021).

Third, nurses’ roles in tackling environmental and climate change issues have received little attention from nurses’ associations, with the exception of environmentally oriented associations such as the Canadian Association of Nurses for the Environment (CANE). Previous studies have reported a lack of clarity from professional organizations on addressing climate change (Nicholas & Breakey, 2017) and a lack of research on nursing interventions intended to mitigate the health effects of climate change (LeClair et al., 2021). In this context, it is important to better define the role of nurses in tackling environmental and climate change issues, to educate the public, nurses and other health professionals about the relationships between nursing practice and climate events, and to provide nurses with tools to help integrate this new role into their daily practice. One solution is to create a national expert committee to develop and disseminate consistent information across the country. For example, the ANHE's Nurses Drawdown, a project which is supported by nursing associations across the globe, has the potential to disseminate universal recommendations for tackling environmental and climate change issues (Nurses Drawdown, 2022; Schenk, 2021). A national expert committee would support the development of guidelines, indicators and incentives. Others have suggested that nursing regulatory bodies could incorporate climate change and sustainability into practice requirements or standards of practice (Dossey et al., 2019); in particular, Leffers (2018) suggests integrating climate change and health into the American Nurses Association's (ANA) Standards of Practice. This could help nurses better understand what problems they are expected to address and what adaptation and mitigation strategies they should use (Nicholas & Breakey, 2017).

Fourth, we must clarify how nursing education can contribute to addressing environmental and climate change issues (Shea et al., 2020); (Teherani, 2017). Five documents analyzed in our review offered recommendations concerning nurses’ education, and previous studies have underlined that nursing educators would like to improve their knowledge on the topic and to be provided with high-quality teaching tools about the health impacts of climate change (Field et al., 2020; Lal et al., 2021). Relatedly, some authors have advocated for a paradigm shift towards a more holistic and sustainable concept of health in nursing education (Anderko et al., 2014; Kurth, 2017; Lilienfeld et al., 2018). For instance, the One Health approach has been associated with several benefits, including increased collaboration between academics, policymakers and citizens to solve problems related to climate change (Zinsstag et al., 2018). This approach could be used to advocate for including nurses in such collaboration around climate change mitigation.

Also, a planetary health perspective could help nurses understand the relationships between human and planetary health (Kalogirou et al., 2020). For instance, the Ecological Planetary Health Model could be used as a framework for the inclusion of climate change education in nursing education curriculum or professional education (Leffers et al., 2017). As an example, *Understanding Climate Change in Nursing Practice*, a guide and educational tool, offers a climate change lesson plan for both clinical and academic settings, and the guide was developed with a planetary health perspective (Radu, 2020). While several isolated initiatives for developing training tools are underway, most address specific health topics (e.g., the e-resource for nursing education on climate-driven vector-borne diseases developed by the Canadian Association of Schools of Nursing). A significant gap remains between nurses’ needs and the training actually available. More research is needed to address ways to integrate climate change issues into nursing education, particularly as curricula are already very full.

### Study recommendations and limitations

Our study provides a list of 37 recommendations that can be used by various stakeholders to clarify and promote what roles nurses might play in addressing climate change and environmental issues. To our knowledge, it is one of the few studies to document the literature published by nursing associations on this subject (Leffers et al., 2017; Lilienfeld et al., 2018). However, this study also has its limitations. First, this narrative review of the literature was not a systematic review and did not focus on scientific papers. Second, the literature search omitted provincial and state-level nursing associations in Canada and the US, with the exception of the province of Quebec. The review focused instead on nationwide nursing associations in Canada and the United States, international nursing organizations, and, as mentioned, Quebec provincial associations. These levels of analysis were chosen for a larger research project on Quebec nurses and climate change. Third, the analysis did not take into account initiatives for specific health issues related to climate change (e.g., Lyme disease). Finally, our review did not include recommendations from webinars, seminars, professional trainings or other oral presentations. Considering the significant role continuing nursing education plays in disseminating innovations, these types of documents may very well be another source of recommendations.

## Conclusion

Climate change is one of the biggest challenges of our time. As highly trusted professionals, nurses have a role to play in tackling climate change. The objective of this article was to document the recommendations of Quebec, Canadian, American and international nursing associations regarding nursing practices to address environmental and climate change issues. Our study identified and summarized 37 recommendations, illustrating the range of actions needed to tackle environmental and climate issues from a nursing perspective. It also brought to light the need to fund nursing innovations that address climate change, the need for such innovations to address health inequities, the need for more nurses’ association initiatives on climate change, and the need to clarify how nursing education can integrate instruction on climate change and related health concerns. As there is a clear need for more research, and since the documents included in this study were recent (most were published in the last five years), we see a great opportunity for intervention research in which nurses implement some of these recommendations into their daily practice. This research could reveal nurses’ perceptions of these recommendations, help define their role in addressing climate change, highlight nurses as assets in mitigating climate change issues, and work towards a better integration of climate change issues into nursing education.
